# Biochemical identification of prepubertal boys with Klinefelter syndrome by combined reproductive hormone profiling using machine learning

**DOI:** 10.1530/EC-22-0537

**Published:** 2023-04-06

**Authors:** Andre Madsen, Anders Juul, Lise Aksglaede

**Affiliations:** 1 Hormone Laboratory, Department of Medical Biochemistry and Pharmacology, Haukeland University Hospital, Bergen, Norway; 2 Department of Growth and Reproduction, Copenhagen University Hospital - Rigshospitalet, Copenhagen, Denmark; 3 International Center for Research and Research Training in Endocrine Disruption of Male Reproduction and Child Health (EDMaRC), Copenhagen University Hospital - Rigshospitalet, Copenhagen, Denmark; 4 Department of Clinical Medicine, University of Copenhagen, Copenhagen, Denmark

**Keywords:** Klinefelter syndrome, early diagnosis, classification, machine learning

## Abstract

**Objective:**

Klinefelter syndrome (KS) is the most common sex chromosome disorder and genetic cause of infertility in males. A highly variable phenotype contributes to the fact that a large proportion of cases are never diagnosed. Typical hallmarks in adults include small testes and azoospermia which may prompt biochemical evaluation that typically shows extremely high follicle-stimulating hormone and low/undetectable inhibin B serum concentrations. However, in prepubertal KS individuals, biochemical parameters are largely overlapping those of prepubertal controls. We aimed to characterize clinical profiles of prepubertal boys with KS in relation to controls and to develop a novel biochemical classification model to identify KS before puberty.

**Methods:**

Retrospective, longitudinal data from 15 prepubertal boys with KS and data from 1475 controls were used to calculate age- and sex-adjusted standard deviation scores (SDS) for height and serum concentrations of reproductive hormones and used to infer a decision tree classification model for KS.

**Results:**

Individual reproductive hormones were low but within reference ranges and did not discriminate KS from controls. Clinical and biochemical profiles including age- and sex-adjusted SDS from multiple reference curves provided input data to train a ‘random forest’ machine learning (ML) model for the detection of KS. Applied to unseen data, the ML model achieved a classification accuracy of 78% (95% CI, 61–94%).

**Conclusions:**

Supervised ML applied to clinically relevant variables enabled computational classification of control and KS profiles. The application of age- and sex-adjusted SDS provided robust predictions irrespective of age. Specialized ML models applied to combined reproductive hormone concentrations may be useful diagnostic tools to improve the identification of prepubertal boys with KS.

## Introduction

Klinefelter syndrome (KS) is the most common sex chromosome disorder affecting approximately 1:660 males (1). Timely diagnosis may be essential to prevent conditions such as osteoporosis, metabolic syndrome, type 2 diabetes, and cardiovascular disease that are associated with increased morbidity and mortality (2, 3). However, the phenotypic spectrum of KS is very wide, and especially during childhood the symptoms are often very subtle, and less than 10% of the expected number are diagnosed before puberty (1). Most patients are diagnosed during infertility workup in adulthood at an age where many years of potential preventive actions are lost.

The hypothalamo–pituitary–gonadal (HPG) hormone axis shows varying activities during postnatal life in healthy subjects; high activity is observed in minipuberty (4), followed by a quiescent period during childhood until pubertal onset following which marked changes in serum concentrations of reproductive hormones are observed until the mature adult phenotype with full reproductive capacity is attained. This means that evaluation of the HPG hormone axis is difficult from 1 to 12 years of age due to the physiologically low hormone activity in healthy subjects. In subjects with KS, serum concentrations of reproductive hormones are abnormal during minipuberty (5) and adult life (6) where the HPG axis is fully active. By contrast, it is difficult to identify abnormal hormone concentrations in individual pre- and peripubertal boys with KS as these are largely overlapping those of controls.

Traditional references in pediatric hormone concentrations are stratified in distinct age intervals, but this crude approach may be sub-optimal for diagnosing specific endocrine conditions. Recent adoption of continuous hormone references using the well-established ‘LMS’ growth curve algorithm provides a clinically useful framework to standardize age- and sex-dependent variance and obtain standard deviation scores (SDS) (4, 7, 8, 9). Briefly, the LMS method accounts for local skewness (L), mean (M), and coefficient of variation (S) to establish continuous normal distributions with age (10).

Supervised machine learning (ML) is an artificial intelligence method that is used for optimizing the interpretation of complex or large datasets. During recent years, ML has increasingly been implemented in the field of medicine as a tool for optimizing diagnostics (11, 12, 13, 14). By bootstrapping and cross-validating training data, the ‘random forest’ algorithm produces several decision trees that ‘vote’ to classify new cases according to the pattern established by the training process (15). A recent publication outlined a ML model to detect KS patients among adult male patients with azoospermia (13).

The aim of this study was to characterize the combined reproductive endocrine profiles of prepubertal boys with KS in relation to those of controls and to develop a novel classification model to predict new cases of KS in the pediatric population. Hereby, we intend to increase the likelihood of detecting, diagnosing, and providing healthcare for KS patients at a much younger age.

## Materials and methods

### Cohort description

Longitudinal, retrospective clinical and biochemical data from a total of 35 visits were obtained from 15 untreated prepubertal boys with 47,XXY KS aged 6.3–13.4 years. Data from routine clinical visits were obtained from patient records from a tertiary center at The Department of Growth and Reproduction, Rigshospitalet, Copenhagen, Denmark. The Klinefelter diagnosis was established prenatally in ten boys and due to psychosocial disorders or learning difficulties in four boys. The reason for diagnosis was unknown in one boy. All boys with KS were prepubertal as defined by testicular volume (TV) < 4 mL, Tanner genital stage G1, and Tanner pubic hair stage PH1. All three puberty measures were used since boys with KS may have entered puberty despite having a TV below 4 mL. Corresponding clinical and biochemical profiles for controls (*n* = 1475) aged 5.8–20.9 years were retrieved from the COPENHAGEN Puberty Study conducted in 2006–2008, as previously described (16). Prepubertal controls in the subgroup (*n* = 729) as defined by TV <4 mL were aged 5.8–13.4 years.

### Clinical evaluation

Body height was measured to the nearest 0.1 cm using a stadiometer. Pubertal evaluation was performed according to the methods by Marshall and Tanner (17). TV was estimated by palpation to the nearest 1 mL the using Prader’s orchidometer (18). In case the volumes of two testes were not equal, the larger testis measurement was used.

### Hormone analyses

Blood samples from boys with KS were drawn as part of the clinical follow-up at routine visits in the outpatient clinic, and all reproductive hormones were analyzed routinely immediately after sampling when ordered by the physician. Blood samples from the controls were stored at −20°C until analysis. All reproductive hormones from both boys with KS and from controls were evaluated in our own laboratory. We performed internal validations and method comparisons and were therefore able to compare measurements, although the methods for measuring sex hormone-binding globulin (SHBG) and anti-Müllerian hormone (AMH) changed during the study period as previously described (19).

Serum concentrations of total testosterone, dehydroepiandrosterone sulfate (DHEAS), 17-hydroxyprogesterone (17-OHP), androstenedione (4A), SHBG, follicle-stimulating hormone (FSH), luteinizing hormone (LH), AMH, and inhibin B were measured. Total testosterone, DHEAS, 17-OHP, and 4A were measured by liquid chromatography-tandem mass spectrometry (LC-MS/MS) with inter-assay coefficients of variation (CVs) ranging from 1.4 to 2.5%, as previously described (7). Limits of quantification for total testosterone, DHEAS, 17-OHP, and 4A were 0.10 nmol/L, 48 nmol/L, 0.19 nmol/L, and 0.18 nmol/L, respectively.

FSH and LH were analyzed by time-resolved immunofluorometric assays (AutoDELFIA; Perkin Elmer) with an inter-assay CV below 5% for both and a limit of detection (LoD) of 0.06 and 0.05 IU/L for FSH and LH, respectively. Until September 2014, serum SHBG was measured by time-resolved immunofluoresence assay (AutoDelfia; Wallac Oy, Turku, Finland) (all controls) with a CV below 6% and an LoD of 0.23 nmol/L, while SHBG from September 2014 and onward was measured by a chemiluminescence immunoassay (Access2, Beckman Coultermodell, Brea, CA, USA) (all patients with KS) with a CV below 5% and an LoD of 0.35 nmol/L. All Access2-derived SHBG results were factored to corresponding AutoDELFIA SHBG results after internal method comparison. An enzyme-linked immunosorbent assay (ELISA) was used to determine inhibin B (Immunotech, Beckman Coulter, Marseilles, France). The LoD and inter-assay CV for inhibin B were 3 pg/mL and 11%, respectively. AMH was measured by AMH-ELISA (Immunotech) until 2015 (all controls) and by chemiluminescence immunoassay (Access 2, Beckman Coulter, Brea, CA, USA) from 2015 and onwards (all patients with KS). Based on an internal validation, the results of the two methods were comparable. The LoD and inter-assay CV for AMH were 0.14 pmol/L and less than 2.5%, respectively.

### Reference curves

Reference curves for body height and serum concentrations of LH, FSH, inhibin B, AMH, total testosterone, SHBG, A4, 17-OHP, and DHEAS were extrapolated from observations of the controls in the COPENHAGEN Puberty Study and modeled using the ‘LMS’ method provided in the Generalized Additive Model for Location, Scale, and Shape (gamlss) package in R (https://cran.r-project.org/web/packages/gamlss/gamlss.pdf). The LMS method summarizes local skewness (L), mean (M), and coefficient of variation (S) and is universally applied to establish anthropometric growth charts (20, 21, 22). Age- and sex-adjusted SDS for all variables were calculated for the boys with KS and compared with age- and sex-adjusted SDS calculated for the subset of controls that were prepubertal as defined by a TV <4 mL (*n* = 64).

### ML and classification

The ‘randomForest’ and ‘caret’ packages in R were used to randomly split the dataset rows into training (75%) and testing (25%) data frames and generate a random forest prediction model. The proportion of prepubertal boys with KS (*n* = 15 patients) and prepubertal controls (*n* = 64 (only controls with data for all parameters in Table 1 were included)) was kept equal when splitting into training and test subsets. Where possible, individual KS patients' age-adjusted *z*-scores recorded over time were averaged and treated as one observation. When the trained ML model was applied to classify unseen data, predictions and reference classifications were arranged as a confusion matrix from which accuracy was calculated as a measure of classification performance. Predictive values of continuous variables were computed as receiver operating characteristic (ROC) curves using the ‘pROC’ package in R.

**Table 1:** Age- and sex-adjusted SDS for body height and serum concentrations of reproductive hormones in prepubertal controls and in prepubertal boys with KS and ability of single variables to classify KS cases.

	Prepubertal controls (*n* = 121–726)	Prepubertal boys with KS (*n* = 15)	*P*	ROC AUC	Sensitivity	Specificity
LH (SDS)	−0.40 (−1.95 to 1.51)	−1.00 (−2.66 to 0.87)	**<0.05**	69.2%	51.5%	83.0%
FSH (SDS)	0.02 (−1.84 to 1.87)	−0.47 (−2.38 to 0.71)	**<0.05**	65.3%	60.6%	67.6%
Inhibin B (SDS)	−0.36 (−2.18 to 1.61)	−0.34 (−1.86 to 1.61)	0.450	51.9%	75.7%	41.9%
AMH (SDS)	0.1 (−1.63 to 1.86)	0.79 (−1.07 to 2.45)	**<0.01**	72.0%	86.2%	59.2%
Testosterone (SDS)	−0.38 (−1.19 to 0.37)	−0.49 (−6.06 to 0.27)	0.569	46.8%	42.4%	65.3%
SHBG (SDS)	0.14 (−1.67 to 2.26)	−0.06 (−1.59 to 1.18)	0.266	61.0%	30.3%	87.8%
Androstenedione (SDS)	−0.03 (-1.85 to 1.89)	−0.31 (−1.66 to 2.50)	0.350	56.2%	51.5%	63.8%
17-OHP (SDS)	0.03 (−1.34 to 1.82)	−0.58 (−1.31 to 2.03)	**<0.05**	70.2%	60.6%	80.1%
DHEAS (SDS)	−0.12 (−1.85 to 1.70)	−0.92 (−2.45 to 2.15)	**<0.01**	72.2%	70.0%	67.3%
Height for age (SDS)	−0.11 (−1.99 to 1.75)	0.16 (−2.32 to 2.34)	0.497	57.1%	40.6%	80.9%

SDS are presented for 64 prepubertal controls (TV < 4 mL) and 35 observations from 15 prepubertal boys with KS (TV < 4 mL, Tanner G stage 1, and Tanner pubic hair stage 1) in the age interval of 5.8– 13.4 years. For boys with KS, intra-individual observations were averaged prior to calculating reference intervals. The ability of the indicated variables to correctly classify controls and boys with KS was evaluated by binary ROC analyses. Data presented as median and (2.5th−97.5th percentile). *P*-values in bold/italics are <0.05. Sample size of prepubertal controls: LH, FSH, SHBG (*n* = 646); inhibin B (*n* = 291); AMH (*n* = 622); testosterone (*n* = 121); androstenedione (*n* = 160); 17-OHP (*n* = 146); DHEAS (*n* = 165); and height for age (*n* = 726). Sample size of prepubertal boys with KS: *n* = 15 for all measured parameters. For boys with KS, multiple intra-individual measurements over time were averaged prior to comparisons with healthy boys.

The final ML model produced has been made available on R Shiny application portal as a proof-of-concept framework to leverage artificial intelligence-assisted diagnosis (https://andremadsen.shinyapps.io/Klinefelter_ML/).

### Statistical analyses

The native principal component analysis (PCA) function ‘prcomp()’ in R was applied to the SDS profiles of prepubertal controls and boys with KS in the dataset, as previously demonstrated (23). The ‘ggbiplot’ package was used to visualize the dimensionality reduction obtained from PCA as a biplot, as described previously (24). Statistical comparisons were done using the Mann–Whitney *U-*test, and *P*-values were considered significant if *P* < 0.05.

### Ethics

Access to and management of data from patient files from boys with KS was approved by the Danish Patient Safety Authority (no. 3-3013-1376/1/), the Team for Medical Records Research, Centre for Health, the Capital Region of Denmark (R-22031906), and the Danish Data Protection Agency (no. 2015-235 (I-Suite no. 04204) and P-2022-364).

The COPENHAGEN Puberty Study was approved by the local ethical committee (KF 01 282214 and V200.1996/90) and was conducted in accordance with the Second Helsinki Declaration. All participants and their parents signed informed consents.

## Results

### Clinical characteristics of prepubertal boys with KS

**Figure 1:**
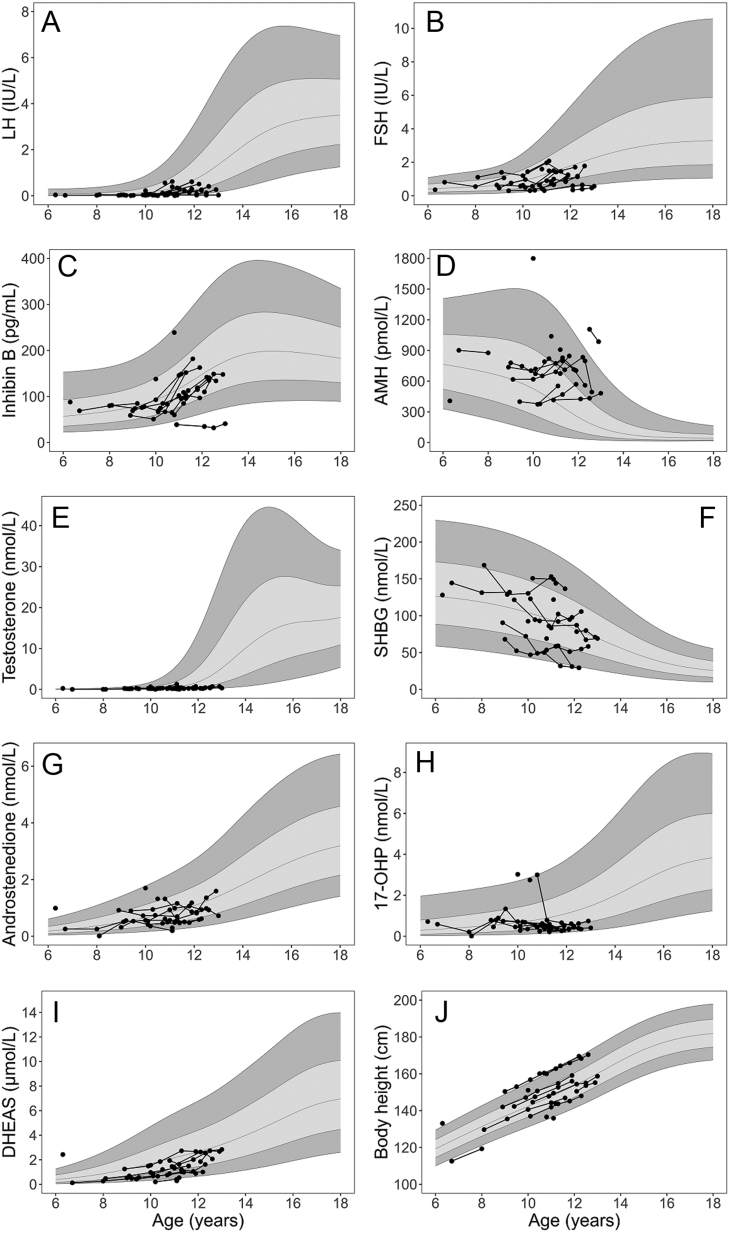
Body height and serum concentrations of reproductive hormones in prepubertal boys with Klinefelter syndrome in relation to normal reference ranges. Measurements of body height and serum concentrations of LH, FSH, inhibin B, AMH, total testosterone, SHBG, A4, 17-OHP, and DHEAS according to chronological age in prepubertal boys with KS are shown in relation to the reference curves based on observations from controls. Lines represent mean (±1 and ±2 SD). Prepubertal boys with KS are indicated with black dots, connected by lines in cases of longitudinal monitoring of the same individual.

Body height and serum concentrations of LH, FSH, inhibin B, AMH, total testosterone, SHBG, A4, 17-OHP, and DHEAS in prepubertal boys with KS according to chronological age are depicted in Figure 1. When comparing to the reference curves, the serum concentrations of total testosterone in prepubertal boys with KS were low and remained low after 10 years of age (Fig. 1E). Serum LH was below mean in all but three boys, and after the age of 11 years all LH concentrations were below mean in the prepubertal boys with KS (Fig. 1A). Likewise, FSH concentrations were generally lower compared to controls but to a lesser extent than LH (Fig. 1B). AMH concentrations in boys with KS were above mean in all but three boys, whereas inhibin B concentrations were within the reference and clustered around mean in all but one boy (Fig. 1C and D). The adrenal hormones were in general low (17-OHP and DHEAS) except A4 that clustered around mean (Fig. 1G, H and I).

Body height was equally distributed through the reference range, and each boy with longitudinal measurements followed his own curve (Fig. 1J).

Age- and sex-adjusted SDS for body height and serum concentrations of LH, FSH, inhibin B, AMH, total testosterone, SHBG, A4, 17-OHP, and DHEAS were calculated for both prepubertal controls and for prepubertal boys with KS (Table 1). When comparing prepubertal controls and prepubertal boys with KS, the serum concentrations of LH, FSH, DHEAS, and 17-OHP were significantly lower in boys with KS, whereas the concentration of AMH was significantly increased in prepubertal boys with KS. No statistical differences between the two groups were observed regarding SDS for total testosterone, inhibin B, SHBG, A4, and body height.

In order to evaluate the feasibility of detecting the KS profile by means of biochemical and clinical data, predictive values of all current variables including body height as well as serum concentrations of LH, FSH, inhibin B, AMH, total testosterone, SHBG, A4, 17-OHP, and DHEAS were assessed by ROC analyses. The ability of each variable to distinguish prepubertal boys with KS from controls is listed as ROC, area under the curve (AUC), and in terms of sensitivity and specificity (Table 1). As evident from the ROC analyses, no singular biochemical or clinical feature provided satisfactory predictive value to detect prepubertal KS in a clinical setting.

### PCA of KS endocrine profile perturbations

**Figure 2:**
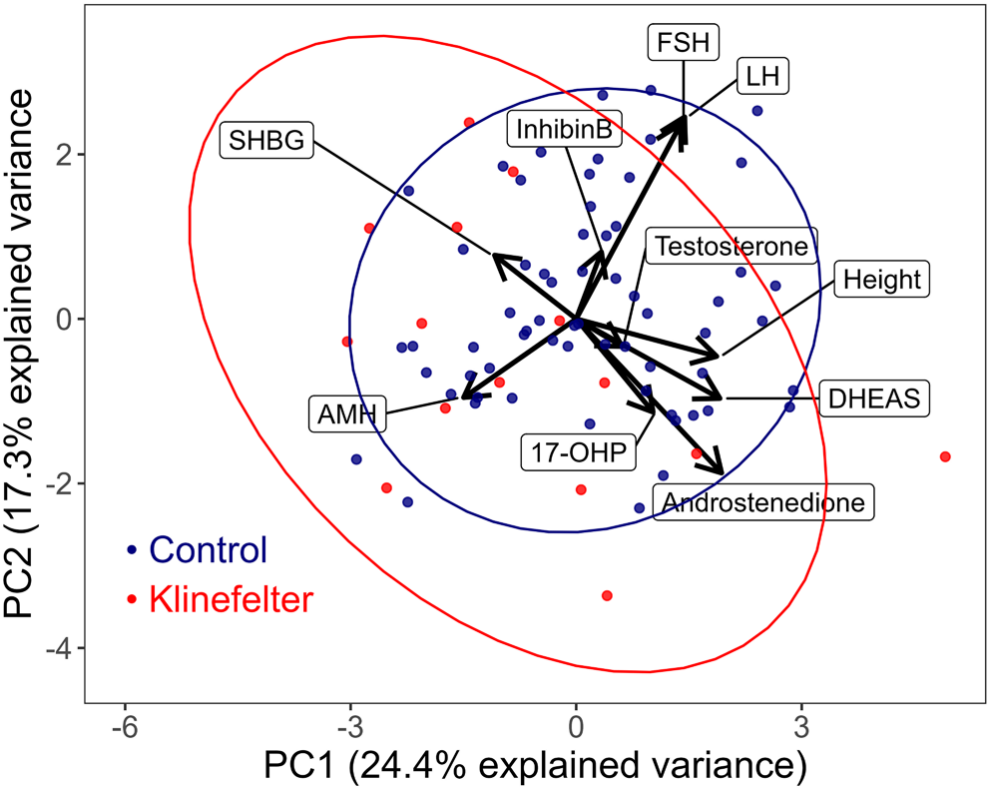
Differing SDS profile in boys with KS compared to controls. PCA biplot indicating the contribution of endocrine and anthropometric variables to dataset variance when comparing controls (blue dots) and boys with KS (red dots). Intra-individual observations were averaged, and clustering of controls and boys with KS was assessed by 1.5 s.d. confidence ellipses. Arrows for each variable (indicated with lines) point in the direction of increasing values of that variable. Horizontal arrow components along the PC1 axis explain slightly more variance than vertical components along the PC2 axis.

PCA was applied to age- and sex-adjusted SDS of prepubertal control boys and prepubertal boys with KS to explore unbiased systematic differences with regard to serum concentrations of LH, FSH, inhibin B, AMH, total testosterone, SHBG, A4, 17-OHP and DHEAS, and body height. Dimensionality reduction by PCA was used to explore potential clusters in the total dataset variance. Variance clusters are detectable by PCA when two or more phenotypes differ systematically across several feature variables, and we hypothesized that this would apply to the current dichotomy of KS and controls. PCs 1 and 2 explained 24.4 and 17.3% of the total dataset variance, respectively. The resulting biplot, visualizing individual observations of KS patients and controls in context of the total dataset variance, largely indicated overlapping endocrine profile clustering (i.e. systematic differences) between prepubertal KS patients and prepubertal controls, and neither PC1 nor PC2 coordinates differed significantly between the two groups (Fig. 2).

### Leveraging ML and biomarker SDS for clinical classifications

The predictive values of the parameters presented in Table 1 to distinguish between KS and controls prompted us to explore a refined prediction model to detect cases of KS. A supervised decision tree ML model was trained to classify ‘KS’ and ‘control’ cases using the SDS profile of serum concentrations of LH, FSH, inhibin B, AMH, total testosterone, SHBG, A4, 17-OHP and DHEAS, and body height featured in Figure 1. Applying the trained ‘random forest’ ML model to classify unseen observations (i.e. 25% of the data not encountered in the training dataset), predictions were made and arranged in a confusion matrix (Table 2). With 10 independent iterations of randomizing which observations were used for training, the model achieved a prediction accuracy of 77.8% (95% CI; 61.1–94.4%) and Cohen's kappa of 0.63 (95% CI; 0.45–0.77) when applied to unseen data. Using the ML model prediction probability score (i.e. decimal scores between 0 (0%, indicating ‘control’) and 1 (100%, indicating ‘KS’) assigned to each new observation), an average ROC AUC of 82.4% (95% CI: 71.1–97.8%) was obtained, thus outperforming the predictive value of any singular variable as declared in Table 1. Optimal classification accuracy was observed when ML prediction scores above 0.31 (30.7%) were labeled as ‘KS’. This cutoff is associated with a sensitivity of 100% (i.e. no false-positive predictions) and a specificity of 88.2%. Lowering of the ML prediction score cutoff markedly reduced the model specificity.

**Table 2:** Supervised ML classification performance.

Prediction	Reference	
	Control	Klinefelter
Control	15	1
Klinefelter	0	2

Representative confusion matrix for ML classifications assigned to prepubertal controls and prepubertal boys with KS. The matrix was obtained from applying the trained ML algorithm to unseen test data, corresponding to 20% of the initial dataset where the proportion between boys with KS and controls was kept constant. With 10 independent iterations of training and classifying unseen data, the ML model achieved an average accuracy of 80%. For boys with KS, intra-individual observations were averaged prior to statistical modeling or prediction.

## Discussion

In this study, we describe the clinical and biochemical profiles of prepubertal boys with KS and evaluate a classification model trained to predict KS before puberty. When comparing the prepubertal boys with KS with prepubertal controls of the same age, we found significantly lower LH, FSH, DHEAS and 17-OHP but significantly higher AMH in the boys with KS (age-adjusted SDS, *P* < 0.05). We found no difference regarding total testosterone, inhibin B, A4, and body height (age-adjusted SDS, *P* > 0.05). Dimensional reduction of the prepubertal phenotypes was resolved by PCA and revealed some degree of clustering) (i.e. overall differences) in the KS endocrine profiles compared to that of controls. Leveraging all the available feature variables above to define the prepubertal KS phenotype, we trained a decision tree-based ML model to differentiate between KS patients and age-matched controls. When applying the ML model to predict (i.e. binary classification of control or KS status) unseen data, the obtained ML model achieved a prediction accuracy of 78% (95% CI; 61–94%) and Cohen's kappa of 0.65 (95% CI; 0.45–0.77), thus outperforming the predictive value of any singular variable (Table 1).

Although KS is recognized as the most frequent genetic cause of hypogonadism and infertility in males, the heterogeneous phenotype comprising this condition makes it notoriously difficult to diagnose, particularly before puberty. Nevertheless, it has been shown that already during infancy and childhood, boys with KS may present with low testosterone (5), and timely diagnosis may therefore be essential for the prevention or reduction of future morbidity. It has been shown that testosterone replacement therapy (TRT) in adults with KS may have positive effects on metabolic health, body composition, and bone mineral density (25). Equally, studies in infants and children with KS indicate that early TRT may improve body composition (26) and thereby potentially reduce a future risk of metabolic syndrome and type II diabetes. In addition, early diagnosis may improve the level of everyday adaptive functioning and quality of life.

In previous studies of prepubertal boys with KS, a normal or even a low LH was found despite low testosterone (27, 28). This is in line with our finding of low LH combined with normal total testosterone and may indicate some altered pituitary–gonadal interaction before puberty. We found normal inhibin B but elevated AMH and lower FSH. In previous studies, a low inhibin B and elevated AMH and FSH were found (27, 29). We have previously shown that the expected decline in AMH during puberty is postponed in boys with KS, and it was speculated that this was due to a delayed puberty as compared with the reference population (30). However, in the present study, the comparison was made between age- and sex-adjusted SDS from prepubertal controls and prepubertal boys with KS. The normal pubertal decline in AMH coincides with increasing concentrations of intratesticular testosterone, and it has been shown that Sertoli cell maturation and thus the expression of androgen receptors are induced by high intratesticular testosterone concentrations (31). Our finding of a postponed decline in AMH could therefore also be the result of impaired intratesticular testosterone concentrations in these boys. The classical description of KS includes tall stature, and growth has been shown to accelerate before the onset of puberty from the age of 5 years in boys with KS (32). In this study, there was no difference in height SDS between the prepubertal boys with KS and the prepubertal controls, and the included boys seemed to follow their own curve. However, we did not have data on parental height, and we therefore do not have information on potential deviation from target height.

The main strength of this pilot study is the use of clinical and biochemical data obtained from well-characterized cohorts of controls and boys with KS. All hormones were measured in our own laboratory, and androgens were measured by LC-MS/MS, which is essential especially in prepubertal boys. In addition, the use of age-adjusted SDS allowed for characterization by PCA and for comparison across age. A limitation of the study is the small number of boys with KS included.

The current rationale to leverage age- and sex-adjusted biomarker SDS scores was previously explored as a reference framework for steroid hormones (7, 8, 33). In line with the stated baseline characteristics for control and KS observations, PCA provided a systemic exploration of biochemical differences between controls and boys with KS. Although the control and KS clinical profiles were not arranged as disjoined clusters in the PCA biplot or satisfactorily distinguishable by singular variable ROC analyses, we proposed that a decision tree-based ML approach could achieve an acceptable classification accuracy. In line with this, a recent study evaluated a model based on age, TV, semen volume, height, and concentrations of LH and FSH for identifying patients with KS among patients with azoospermia (13). The model was based on data from a total of 1339 azoospermic patients including 345 patients with KS, and the sensitivity of that model was 100% and the specificity >93%, which was significantly better compared to 18 expert clinicians.

ML applied to biochemical data was able to make valid predictions to accurately classify cases of KS in this retrospective pilot study, but the model should be verified with larger datasets prior to clinical implementation. Notably, the current random forest classification model outperformed all surveyed individual biomarkers and may yet be improved by the addition of other anthropometric and pertinent biochemical markers. The direct clinical utility of the current model may be limited, but this model shows that the reproductive hormone profile can discriminate boys with KS from controls already before puberty. The variables included in this model may be modified in a future model to better fit in a screening scenario in primary healthcare where a complete biochemical evaluation including reproductive hormones may not be available.

The final ML model produced from the observations of prepubertal KS patients and controls has been made available as a proof-of-concept framework to leverage artificial intelligence-assisted diagnosis. However, the actual predictions made from the model should not be regarded as medical advice. Although there is no precise way to determine metrics of 'model overfitting', we expect that the current decision tree-based ML model was primed to capture the few available observations of KS. Over-adaption of statistical models to fit small sample sizes is a universal problem in biostatistics and data science.

In conclusion, we propose that the LMS reference curve framework and application of clinically relevant age- and sex-adjusted SDS enable computational ML prediction models to complement clinical investigations in pediatric endocrinology.

## Declaration of interest

The authors have no conflicts of interest.

## Funding

This study was supported by Rigshospitalet http://dx.doi.org/10.13039/501100005111s Research council (AJ and LA).
